# Evidence for Positive Selection in Putative Virulence Factors within the *Paracoccidioides brasiliensis* Species Complex

**DOI:** 10.1371/journal.pntd.0000296

**Published:** 2008-09-17

**Authors:** Daniel R. Matute, Lina M. Quesada-Ocampo, Jason T. Rauscher, Juan G. McEwen

**Affiliations:** 1 Department of Ecology and Evolution, University of Chicago, Chicago, Illinois, United States of America; 2 Department of Plant Pathology, Michigan State University, East Lansing, Michigan, United States of America; 3 Department of Biology, University of Puerto Rico–Río Piedras, San Juan, Puerto Rico; 4 Corporación para Investigaciones Biológicas (CIB), Medellín, Colombia; 5 Universidad de Antioquia, Medellín, Colombia; University of California Berkeley, United States of America

## Abstract

*Paracoccidioides brasiliensis* is a dimorphic fungus that is the causative agent of paracoccidioidomycosis, the most important prevalent systemic mycosis in Latin America. Recently, the existence of three genetically isolated groups in *P. brasiliensis* was demonstrated, enabling comparative studies of molecular evolution among *P. brasiliensis* lineages. Thirty-two gene sequences coding for putative virulence factors were analyzed to determine whether they were under positive selection. Our maximum likelihood–based approach yielded evidence for selection in 12 genes that are involved in different cellular processes. An in-depth analysis of four of these genes showed them to be either antigenic or involved in pathogenesis. Here, we present evidence indicating that several replacement mutations in *gp43* are under positive balancing selection. The other three genes (*fks*, *cdc42* and *p27*) show very little variation among the *P. brasiliensis* lineages and appear to be under positive directional selection. Our results are consistent with the more general observations that selective constraints are variable across the genome, and that even in the genes under positive selection, only a few sites are altered. We present our results within an evolutionary framework that may be applicable for studying adaptation and pathogenesis in *P. brasiliensis* and other pathogenic fungi.

## Author Summary

The fungus *Paracoccidioides brasiliensis* is the causative agent of paracoccidioidomycosis, a severe pulmonary mycosis that is endemic to Latin America, where an estimated 10 million people are infected with the fungus. Despite the importance of this disease, we know little about the ecological and evolutionary history of this fungus. Here, we present a survey of genetic variation in putative virulence genes in *P. brasiliensis* in what constitutes the first systematic approach to understand the molecular evolution of the fungus. We used a population genetics approach to determine the role has natural selection played in the coding genes for proteins involved in pathogenesis. We found that nonsynonymous mutations are more common in genes that code for virulence factors than in housekeeping genes. Our results suggest that positive selection has played an important role in the evolution of virulence factors of *P. brasiliensis* and is therefore an important factor in the host–pathogen dynamics. Our results also have implications for the possible development of a vaccine against paracoccidioidomycosis, since *gp43*—the main vaccine candidate—has a high level of polymorphism maintained by natural selection.

## Introduction

The neutral theory of evolution states that most evolutionary change at the molecular level is caused by the fixation of neutral alleles through random genetic drift [1]. Nonetheless, it is the impact of natural selection on genomic evolution that is of interest if we wish to understand patterns of adaptive evolution by distinguishing between selectively neutral and non-neutral evolutionary change, and relate this change to the biology and history of the organism. The arms race between hosts and their pathogens is a particularly useful system for relating potentially non-neutral evolutionary change to the biology and history of the organisms [2],[3] because of the role natural selection plays in maintaining or fixing different alleles in both host and pathogen populations [4].

Human-fungal interactions provide a privileged system to study the impact of natural selection on the genome of fungal pathogens. *Paracoccidoides brasiliensis* is the etiological agent of paracoccidioidomycosis (PCM), a human systemic mycosis of importance in Latin America [5]. It is endemic to an area extending from Mexico to Argentina, and infects an estimated 10 million people [6]. Recently, the existence of genetically distinct evolutionary lineages within *P. brasiliensis* was demonstrated through analysis of DNA sequence data for multiple genes [7],[8]. These groups are currently designated S1 (species 1), PS2 (phylogenetic species 2), PS3 (phylogenetic species 3) and Pb01. Additional support for these lineages comes from variation in virulence and expression levels of antigenic proteins previously found between *P. brasiliensis* isolates which are now known to belong to S1 and PS2 groups [9]. The recent publication of genomic sequences in the form of expressed sequence tag (EST) databases for several isolates of the different genetic groups of *P. brasiliensis* [10],[11],[12] and the closely-related species *Histoplasma capsulatum* (*Ajellomyces capsulatum*) (unpublished results) presents an opportunity to investigate the role that natural selection may have played in shaping the molecular evolution of the *P. brasiliensis* genome. Comparative studies between the *P. brasiliensis* genetic groups and *H. capsulatum* can be useful to understand host-pathogen evolution, especially in the genes encoding pathogenesis-related proteins which are likely to evolve in response to selective pressure from the host's immune system.

Detecting natural selection at the molecular level requires statistical tests that distinguish the genomic signature of selection from that of neutral mutation and genetic drift alone. Positive selection is inferred when ω [13] (the ratio of non-synonymous (dN) to synonymous (dS) mutations between species) exceeds 1. Positive directional selection occurs when successive amino acid changes make a protein better adapted in a particular biological context, and as a result the changes will tend to be fixed in future lineages. Positive diversifying selection occurs when multiple phenotypes in a population are favored, resulting in an overall increase of the genetic diversity within the species [14],[15]. Several likelihood methods have also been developed to detect deviations from neutral expectation. Under an infinite-sites model, the level of DNA polymorphism within a species is proportional to the amount of divergence at that locus among closely related species [16]. Deviations from this model form the basis for various tests of natural selection, such as the HKA test [17], and the M-K test [18]. Moreover, likelihood methods that allow ω to vary among the branches in a phylogeny, as well as between codons, have been proposed [19],[20],[21],[22],[23]. Using such methods, several genes involved in defense systems and immunity, as well as toxic protein genes, have been shown to be under diversifying or positive directional selection [24],[25],[26],[27].

In this study, we sought to understand the molecular evolution of candidate genes associated with *P. brasilensis* fungal pathogenesis, which are hypothesized as being under positive selection due to their role in the host-pathogen immune system interaction. Thirty-two putative virulence factors described in previous studies [9],[10],[11],[12],[28] were selected from two available EST databases [10],[11]. In addition, we randomly selected 32 putative housekeeping genes without known antigenic or virulence properties to be used as controls. Orthologous sequences from *P. brasiliensis* and *H. capsulatum* were tested for positive selection by means of the Nei and Gojobori method [29], which calculates the average ratio across all amino acid sites. For those genes that showed some evidence of positive selection we obtained sequences from the three lineages of *P. brasilensis* and used maximum likelihood methods to identify amino acid residues on which positive selection has acted [30]. Our results suggest that positive selection has indeed played an important role in the molecular evolution of virulence factors of *P. brasiliensis*.

## Materials and Methods

### *P. brasiliensis* isolates

The *P. brasiliensis* strains used in this study were described previously [7]. The sample included individuals from four biotypes recognized for *P. brasiliensis*: Pb01 (n = 1), S1 (n = 46), PS2 (n = 6) and PS3 (n = 23) and was representative of six endemic areas for paracoccidiodomycosis. We used sequences from GenBank under accession numbers DQ003724 to DQ003788 as well as new sequences obtained by methods previously described [7]. Briefly, total DNA was extracted from the yeast culture with protocols using glass beads [31] or maceration of frozen cells [32]. PCR primers and conditions were as previously reported [7]. The new sequences were deposited in GenBank under accession numbers EU283774 to EU283809.

### Selection of putative virulence factors

Molecular genetic tools are still not fully developed for *P. brasiliensis*, hindering studies that seek to molecularly define genetic factors involved in *P. brasiliensis* pathogenesis. For the dimorphic fungi, a virulence factor has been functionally defined as a gene product that has an effect on the survival and growth of the organism in its mammalian host but is not essential for growth of the parasitic phase *in vitro* [33]. Nevertheless, the study of virulence genes *sensu* Rappleye and Goldman in isolation [33] does not provide full picture of their evolution, because the molecular basis of virulence involves complex networks that comprise many classes of genes. We focused on all the genes proposed to have an impact on the virulence of *P. brasiliensis*. Table S1 lists the genes that, following genomic analysis in *P. brasiliensis*, were considered as potential virulence factors and, as such, candidates for this survey [10],[11],[12]. For a gene to be included in this analysis, it had to fulfill three conditions: (*i*) to have been reported as a putative virulence factor in the previous literature [9],[10],[11],[12],[28], (*ii*) to be present in the three analyzed databases (two ESTs databases from *P. brasiliensis* and the genome of *H. capsulatum*), and (*iii*) have been demonstrated to be a virulence factor or be an ortholog of a proven virulence factor and have a high homology with it (<1E-10). Fifty percent (32 genes) of the 64 initial candidates fulfilled our requirements and were analyzed to detect positive selection.

### Tests for positive selection: *H. capsulatum* vs. *P. brasiliensis*

#### Data retrieving and alignment

Gene sequences were obtained from the National Center for Biotechnology Information (NCBI). The genome sequences from *H. capsulatum* and the EST sequences of *P. brasiliensis* were obtained from the EMBL database (http://www.ebi.ac.uk/Databases/nucleotide.html) as of October 13, 2007. BLAST programs were obtained from the NCBI and run locally (Table S2). The two EST databases used in this study [10],[11] include genes expressed in the yeast phase of *P. brasiliensis*. These databases were compared with the Bastos EST database [12] and early versions of the genome sequence of *P. brasiliensis* Pb18 (unpublished results) to verify that we were working with high quality EST sequences. The sequences were visually checked and edited to avoid frame shift mutations. No false polymorphisms due to sequencing errors were found in the sequences. The orthology of the genes was assessed by using the preliminary version of the *P. brasiliensis* genome (http://www.broad.mit.edu/annotation/genome/paracoccidioides_brasiliensis/MultiHome.html).

Housekeeping genes were selected from the *P. brasiliensis* available sequences in the Gen Bank by using a PERL script, which randomly selected thirty-two genes that did not present any annotation related to virulence or antigenicity.

Alignments of the sequences of the putative virulence factors and housekeeping genes were generated with MUSCLE [34], and the quality of the alignment was assessed with MacClade [35].

#### dN/dS calculation and Z-tests

**Figure 1:**
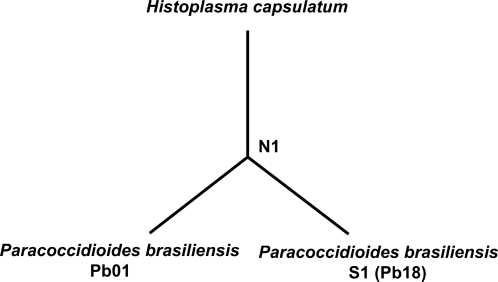
The phylogeny of *H. capsulatum*, *P. brasiliensis* Pb18, and *P. brasiliensis* Pb01 .N1 is the common ancestor of the three branches of the tree.

Using a distance-based Bayesian method, the ancestral sequences were reconstructed (i.e. the common ancestor of the three branches of the tree (N1 in Figure 1)), using the Ancestor software [36] for each gene in the dataset. The predicted sequence of each ancestral state was given a probability, with a 95% or higher cut-off. To test for positive selection we calculated the dN and dS values for each branch of the phylogeny (Figure 1) using the random effect likelihood method of Pond and Frost [37],[38], available in HyPhy [38]. The distance from the common ancestor of the last common ancestor of the two *P. brasiliensis* groups was calculated using an optimal model of nucleic acid selection. Similar results were obtained with other models (HKY85, TN93, and REV).

Additionally, we estimated the dS and dN variances: Var(dS) and Var(dN), respectively. With this information, we calculated dN/dS and tested the null hypothesis of no selection (H0: dN = dS) versus the positive selection hypothesis (H1: dN>dS) using the Z-test: Z = (dN−dS)/√(Var(dS)+Var(dN)). Z tests calculations were performed using the MEGA software [39],[40].

#### Mutational saturation dynamics

**Figure 2:**
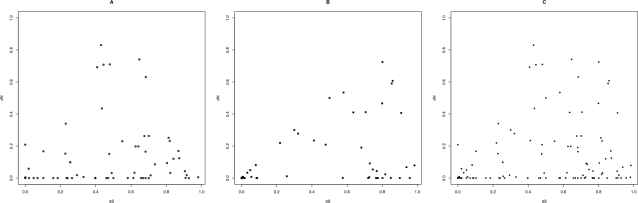
Observed nonsynonymous differences per site (dN) and synonymous differences per site (dS) in pairwise comparisons for three different partitions of genes. A. Putative Virulence factors. B. Randomly selected controls. C. Both groups of genes analyzed altogether.

To examine the relative degree of mutational saturation in non-synonymous and synonymous substitutions in our dataset, we plotted the number of non-synonymous nucleotide differences between the two *P. brasiliensis* groups and the common ancestor against the number of synonymous nucleotide differences for both sets of genes (housekeeping and virulence factors) (Figure 2). Additionally, we fitted a linear model (with functional form dN = A(dS)+B) and a model involving a square term dN = (A(dS)^2^+BdS+C) to the data by the method of least squares [41]. All the statistical analyses were performed with R.

#### M-K tests

M-K tests [18] between the *P. brasiliensis sensu lato* and *H. capsulatum*, using the aligned regions previously sequenced as well as sequences retrieved from GenBank, were calculated using the DNASP analysis program [42].

### Codon-Based Likelihood Analyses within *P. brasiliensis*

To validate our results, we selected a smaller subset of genes that had demonstrated to be under positive selection pressures and for which population datasets were available. The only genes that fulfilled these characteristics were *gp43*, *p27*, *fks* and *cdc42*. In this set of sequences we searched for evidence of positive selection using the CODEML program of the PAML package (version 4) [22],[30] by using **s**everal likelihood-based tests. For each test, equilibrium codon frequencies were estimated from the average nucleotide frequencies at each codon position, amino acid distances were assumed to be equal, and the transition/transversion ratio (κ) was estimated from the data. For all other parameters, we used the default settings described by Yang and Bielawski [30]. Given the observed intraspecific variability, the lack of homoplasy found in individual gene trees, and the phylogenetically recognized groups, we assumed linkage between colinear sites (i.e., there was no recombination within each data set).

To determine which model best fit the data, likelihood ratio tests (LRTs) were performed by comparing the differences in log-likelihood values (LRT = −2lnL) between two models using a χ^2^ distribution, with the number of degrees of freedom equal to the difference in the number of parameters between the models. We used six models implemented in PAML [13],[22],[30] to test for the presence of sites under positive selection (ω>1). The one-ratio model (M0) assumes one ω for all sites. The neutral model (M1) assumes two classes of sites in the protein: the conserved sites at which ω = 0, and the neutral sites that are defined by ω = 1. The beta model (M7) uses a β distribution of ω over sites: β (p,q), which, depending on parameters p and q, can take various shapes in the 0 to 1 interval. The other three models allow sites with ω>1 and can be considered as tests of positive selection. The selection model (M2) has an additional class of sites compared to the neutral model, in which ω is a free parameter and, as such, can change among residues. The discrete model (M3) uses a distribution with three site classes, with the proportions (p0, p1, and p2) and the ω ratios (ω0, ω1, and ω2) estimated from the data. The beta and ω model (M8) added an extra class of sites to the beta model, estimating the proportion of ω from the data. We used LRTs to make 3 comparisons: to find out whether positive selection has played a role in the molecular evolution of these genes the one-ratio model (M0) was compared with the discrete model (M3) and the neutral model (M1) was compared with the selection model (M2). A third comparison (the beta model (M7) vs. the beta and ω model, M8) [30] was used to identified particular sites in the genes that were likely to have evolved under positive selection by using the Bayesian Empirical Bayes (BEB) analysis previously proposed by Yang [13]. Bayes' theorem was used to estimate the posterior probability that a given site came from the class of positively selected sites [13],[30],[43]. In order to predict potential antigenic determinants for HLA recognition, we used the program SYPFETHI [44].

### Estimation of the Time to the Most Recent Common Ancestor (TMRCA)

To determine whether any of the studied loci presented coalescence times within the *P. brasiliensis* clade (which were older than any other loci) we calculated the Time to the Most Recent Common Ancestor (TMRCA). TMRCAs for S1 and PS2 were estimated based on genetic variation at the eight nuclear loci using the program IM [45]. Estimates of TMRCA do not directly estimate the date of divergence; they provide the timing of coalescence of alleles within a taxon. TMRCA estimates can post- or pre-date the speciation event, and thus can indicate whether the polymorphism in any given gene is older or more recent than the polymorphism in the other genes.

## Results

### Tests for positive selection (dN/dS): *H. capsulatum* vs. *P. brasiliensis*

Thirty-two putative virulence factors fulfilled the requirements for inclusion in this analysis. All the virulence factors showed to be single-copy genes (data not shown, available upon request). To be considered as being under positive selection, these genes had to exhibit a dN/dS ratio larger than 1 and a p-value for the Z-test below 0.05. Table 1 shows the dN/dS ratios for the putative virulence factors and their p-values as determined by using the Z test. According to these criteria, 12 genes were determined to be under positive selection. The dN/dS ratio is correlated to the strength of selection, where values >1 indicate positive selection, and larger values indicate stronger selection. Thirty-two housekeeping genes were randomly selected from the *P. brasiliensis* available sequences by using a PERL script and their dN/dS (and associated Z values) were calculated and were used as source of comparison. None of these genes showed evidence of being under positive selection in the *P. brasiliensis* branches, as illustrated in Table 2.

**Table 1:** Ratio of nonsynonymous to synonomous mutation rate (dN/dS) values for putative virulence factors in the *P. brasiliensis* lineage.

Gene	*P. brasiliensis* Pb18/N1		*P. brasiliensis* Pb01/N1	
	dN/dS	p-value	dN/dS	p-value
*ade2*	0.175	1	0.374	1
***his1***	**2.318**	0.01*	1.482	0.06
*mls1*	0	1	0.005	1
*icl1*	0.375	0.47	0.053	1
*hem3*	0.115	0.12	0.8	0.18
*chs3*	0	1	0.142	0.53
***cst20***	**1.930**	0.047*	0.282	1
***cdc42***	**1.890**	0.04*	0.438	1
***R = asB***	0	1	0	1
***ags1***	0	1	**1.855**	0.042*
*cpn10*	0.195	1	0	1
*groEL*	0.361	0.48	0.117	1
*fgsc A4*	0.385	1	0.385	1
*ssc1*	0	1	0.148	0.93
*hsp70* (mitochondrial)	0	1	0.020	1
*hsp70*	0	1	0	1
*hsp82*	0	1	0	1
***hsp88***	0.047	1	**1.595**	0.05*
*hsp90*	0	1	0.316	1
*mdj1*	0.024	1	0	1
*ura3*	0.039	0.51	3.215×10^−11^	1
***fas2b***	**1.980**	0.05*	**2.160**	0.03*
***sod1***	0.060	0.27	**2.654**	0.02*
*ure1b*	0	1	0.064	1
*tsa1*	0.062	1	**1.612**	0.045*
*gas1*	3.980	0.02	0	1
*asp*	0/0	1	0/0	1
***mnn5***	0	1	**5.147**	0.01*
*tcp1*	0.309	1	0.117	1
***fks***	0/0	1	**23.041**	0.001*
***p27***	0.388	1	**1.699**	0.043*
***gp43***	**1.478**	0.01*	**3.100**	0.02*

dN/dS values are shown for the branches that lead towards *P. brasiliensis* groups as showed in Figure 1. dN/dS ratios are correlated with the strength of selection, where values >1 indicate positive selection, and larger values indicate stronger selection. The P value associated to each dN/dS ratio represents the significance of the Z-test for each branch. Genes that had dN/dS value above 1 and its Z-value was significant (<0.05) were considered under positive selection are marked with ^*^.

**Table 2:** dN/dS values for a set of randomly selected genes not related to pathogenesis in the *P. brasiliensis lineage.*

Gene	*P. brasiliensis* Pb18/N1		*P. brasiliensis* Pb01/N1	
	dN/dS	p-value	dN/dS	p-value
*14-3-3*	0	1	0	1
*cyr1*	0.007	1	0.005	1
*adh*	0	1	0	1
*pepN*	0.08	1	0.993	0.3
*atp-synt_B*	0	1	0	1
*erg6*	0	1	0	1
*Calnexin*	0	1	0	1
*cts1*	0	1	0	1
*CLPA*	0.57	0.25	0.436	0.26
*cox15*	0.692	1	0	1
*cox17*	0	1	0	1
*cox23*	0	1	0	1
*cox8*	0.521	0.28	0.92	0.18
*cox11*	0	1	0	1
*RibH*	0	1	0	1
*Glycos_transf_2*	0.629	0.18	0.281	0.17
*eno*	0.697	1	0.697	1
*Fer4*	0	1	0	1
*FBP_aldolase_IIA*	0	1	0	1
*Gp_dh_N*	0	1	0	1
*hyd2*	0	1	0	1
*l10*	0	1	0	1
*L-Dopa*	0.583	1	0	1
*nag*	0	1	0	1
*oxa1*	0.9	1	0	1
*pet100*	0	1	0	1
*phb1*	0	1	0.559	1
*pra*	0	1	0	1
*sco1*	0	1	0.231	1
*sep1*	0.125	1	0.46	1
*zip*	1	0.13	0.864	0.16
*tub1*	0.011	1	0.642	0.42

Conventions are explained in Table 1.

### Mutational saturation

A possible explanation for the high proportion of genes under positive selection is that the high proportion of virulence factors showing significantly higher dN/dS are partly artifacts caused by the methods used to estimate the number of non-synonymous and synonymous mutations [46]. Such an explanation would require saturation to occur faster in synonymous than in non-synonymous sites, i.e., the number of non-synonymous nucleotide differences should be a concave function of the number of synonymous nucleotide differences [41]. We plotted the number of non-synonymous nucleotide differences between the two groups of *P. brasiliensis* and their common ancestor, against the number of synonymous nucleotide differences (Figure 2). No differences were found between the linear and the quadratic models, neither for virulence factors (LRT = 2.134, p = 0.144), nor the housekeeping genes (LRT = 0.112, p = 0.7378), nor for the pooled data (LRT = 1.631; p = 0.2015) indicating that the lineal model is more appropriate to explain the relationship between dN and dS. Therefore, mutational saturation is not responsible for the elevated dN/dS ratios observed in the virulence factors. Similar comparisons were performed including *H. capsulatum*: one virulence factor (*ags1*) and housekeeping gene (*Gp_dh_N*) were found to be under positive selection in the branch that leads towards *H. capsulatum* (data not shown).

Another possibility is that sequencing errors had inflated dN values. Such errors could artificially increase the significance level of the dN/dS test because they would tend to elevate the number of non-synonymous mutations. However, sequencing errors should also elevate the proportion synonymous mutations and missense mutations. If sequencing errors had, indeed, increased dN, then a large proportion of points in Figure 2 should be located in the upper-left region of the plane. Because no such pattern is observed in Figure 2, we consider this explanation unlikely.

Detection of positive selection by several computing packages program is “reliable” but “conservative” [19],[30],[47] when few sequences are used. Increased accuracy and power are most easily gained with more sequences [19],[30]. Therefore, to further validate our methods and distinguish between directional and diversifying selection, we selected a subset of genes. We choose from among the 12 genes that showed both evidence for positive selection and had more than 25 sequences of *P. brasiliensis* in GenBank, then reapplying population genetics analysis to these genes. From the 12 genes listed in Table 1, four were selected to be analyzed more in-depth: *gp43*, *p27*, *cdc42* and *fks*.

### M-K tests

For the gp43 case, the M-K test yielded no significant results between *H. capsulatum* and *P. brasiliensis* (Fischer's exact test, P = 0.40, Table 3). M-K tests were significant for p27, cdc42 and fks (*p27*: P = 0.043594; *cdc42*: P = 0.000993; *fks*: P = 0.000017; Table 3) when *H. capsulatum* was used as an outgroup.

**Table 3:** McDonald-Kreitman tests of neutrality.

	Fixed between *H.capsulatum* and S1		Polymorphic Within species		Fisher's exact test. P-value
	Syn.	Non-Syn.	Syn.	Non-Syn.	
*cdc42*	125	119	11	0	<0.001
*fks*	40	90	14	2	<0.001
*gp43*	1	7	5	13	0.6279
*p27*	35	22	8	0	0.0436

### Codon-Based Likelihood Analyses within *P. brasiliensis*

#### *gp43*

DNA sequences were obtained from 77 *P. brasiliensis* individuals that yielded twenty-six unique alleles in the exon 2 region of *gp43*. A total of 29 polymorphic sites and 33 mutations, including 8 singleton and 21 parsimony informative sites, were found among the *gp43* alleles (π_S1_ = 0.00571; π_PS2_ = 0.00206; π_PS3_ = 0.00031). Eight silent and twenty-five replacement substitutions were found, where the majority (75.7%) of non-synonymous differences occurred as singletons. No insertion-deletions were found.

Log-likelihood values and parameter estimates under each model are listed in Table 4. Selection models provided a significantly better fit to the data than the neutral models (Table 5); comparisons of M2 versus single-ratio and neutral models yielded LRT values of 18.106 (df = 2, P<0.0001) and 9.14 (df = 2, P = 0.0103), respectively. Likewise, tests between beta (M7) and ω (M8) models strongly supported positive selection (LRT = 18.64, P<0.0001). We found evidence of variation in ω among lineages, as well as substantial variation in ω between sites in the data set. The free-ratio model (M3) was compared with a model that assumes a constant ω across all lineages (M0) by performing LRTs. We could not reject M0 for any of the genes except *gp43*. Using the one-ratio model (M0), the average value of ω for the *gp43* gene was 1.168 - significantly higher than for any of the housekeeping genes [48]. The values of the parameters under the discrete model (M3) indicated that 59.3% of the sites in the *gp43* gene were under purifying selection (ω = 0), whereas 37.07% belonged to a site class with ω = 1.63, and 3.6% had an ω equal to 18.53, indicating that the two latter classes are under positive selection.

**Table 4:** Likelihood values, parameter estimates, and sites under positive selection as inferred under the six proposed models applied to each of the four loci.

	Model	lnL	Parameter Estimate	dN/dS	Selected sites			
**GP43**	One-ratio (M0)	−971.307	1.168	1.168	None			
	Neutral (M1)	−975.791	p0 = 0.559996 w0 = 0.0	0.44	Not allowed			
			p1 = 0.44004 w1 = 1.00					
	Selection (M2)	−966.738	p0 = 0.43329 w0 = 0.00	1.2303	231 V**	241 S**	266 I+	296 G+
			p1 = 0.52265 w1 = 1.00		335 P*	336 L+		
			p2 = 0.04406 w2 = 16.20255					
	Free-ratio (M3)	−965.824	p0 = 0.58630 w0 = 0.00	1.2628	218 S**	225 E**	226 D**	229 H**
			p1 = 0.37715 w1 = 1.58443		231 V**	241 S**	248 P**	251 T**
			p2 = 0.03655 w2 = 18.19716		260 T**	265 Y**	266 I**	296 G**
					335 P**	330 S**	348 K**	360 K**
					363 L**	374 E**	376 G**	
	Beta (M7)	−976.065	p = 0.005 q = 0.00829	0.375	Not allowed			
	Beta+w (M8)	−966.745	p0 = 0.95493 p = 0.03212	1.2303	231 V**	241 S**	266 I+	296 G+
			q = 0.02627		335 P*	336 L+		
			p1 = 0.04507 w = 15.93096					
**FKS**	One-ratio (M0)	−869.645	0.1136	0.1136	None			
	Neutral (M1)	−869.645	P0 = 1.00000 wo = 0.11334	0.1136	Not allowed			
			P1 = 0.00000 w1 = 1.00000					
	Selection (M2)	−869.645	P0 = 1.00000 wo = 0.11324	0.1136				
			P1 = 0.00000 w1 = 1.00000					
			P2 = 0.00000 w2 = 3.00000					
	Free-ratio (M3)	−869.645	p0 = 0.03490 wo = 0.11330	0.1136	None			
			p1 = 0.91236 w1 = 0.11324					
			p2 = 0.05274 w2 = 0.11328					
	Beta (M7)	−869.645	p = 12.68894 q = 99.00000	0.1136	Not allowed			
	Beta+w (M8)	−869.645	p0 = 1.00000 p = 12.73125					
			q = 99.00000					
			(p1 = 0.00000) w = 1.00000					
**CDC42**	One-ratio (M0)	−1151.0403	0.1136	0.39594	None			
	Neutral (M1)	−1151.429	P0 = 0.6041 wo = 1.000	0.39594	Not allowed			
			P1 = 0.3959 w1 = 0.00000					
	Selection (M2)	−1150.091	P0 = 0.7645 wo = 0.58454	3.12653				
			P1 = 0.2227 w1 = 1.00000					
			P2 = 0.01283 w2 = 46.95373					
	Free-ratio (M3)	−1150.3119	p0 = 0.8300 wo = 0.46384	1.93988	None			
			p1 = 0.1464 w1 = 0.46384					
			p2 = 0.0246 w2 = 27.5156					
	Beta (M7)	−1179.7537	p = 0.05 q = 0.604263	0.365995	Not allowed			
	Beta+w (M8)	−1178.436	p0 = 1.00000 p = 12.73125	0.396092				
			q = 85					
			(p1 = 0.05) w = 1.00000					
**P27**	One-ratio (M0)	−1341.1883	0.1136	0.506987	None			
	Neutral (M1)	−1341.1883	P0 = 0.500 wo = 0.01397325	0.506987	Not allowed			
			P1 = 0.500 w1 = 1.000000					
	Selection (M2)	−1341.1883	P0 = 0.3333 wo = 0.000	2.0047				
			P1 = 0.014195 w1 = 0.3333					
			P2 = 0.05 w2 = 5.0					
	Free-ratio (M3)	−1341.1883	p0 = 0.3333 wo = 0.01711	0.05133	None			
			p1 = 0.3333 w1 = 0.03422					
			p2 = 0.3333 w2 = 0.10267					
	Beta (M7)	−1341.1883	p = 1.000 q = 1.000	0.5	Not allowed			
	Beta+w (M8)	−1341.1883	p0 = 0.6667 p = 1.000	0.555556				
			q = 2.00 w = 1.000					
			(p1 = 0.667) w = 1.000					

Amino acid sites inferred to be under positive selection with a probability. lnL: log-Likelihood

>99% are marked with a **, more than 95% with a * and more than 75% with a +.

**Table 5:** Likelihood ratio statistics of different models.

Locus	Comparison	Df	lnL	X2 Critical value (1%)
**GP43**	One-ratio (M0) vs. Discrete (M 3)	4	10.966608	9.21
	Neutral (M 1) vs. Selection (M 2)	2	18.10675	13.28
	Beta (M 7) vs. Beta+w (M 8)	2	18.641266	9.21
**FKS**	One-ratio (M0) vs. Discrete (M 3)	4	0	9.21
	Neutral (M 1) vs. Selection (M 2)	2	0	13.28
	Beta (M 7) vs. Beta+w (M 8)	2	0	9.21
**CDC42**	One-ratio (M0) vs. Discrete (M 3)	4	1.4568	9.21
	Neutral (M 1) vs. Selection (M 2)	2	2.676	13.28
	Beta (M 7) vs. Beta+w (M 8)	2	2.6354	9.21
**P27**	One-ratio (M0) vs. Discrete (M 3)	4	0	9.21
	Neutral (M 1) vs. Selection (M 2)	2	0	13.28
	Beta (M 7) vs. Beta+w (M 8)	2	0	9.21

Twice the difference in log likelihood ratio between a null model and an alternative model was compared with a χ^2^ distribution in order to test whether an alternative model fits the data better than the null model. Df: Degrees of Freedom; LRT: Likelihood Ratio Test.

**Figure 3:**
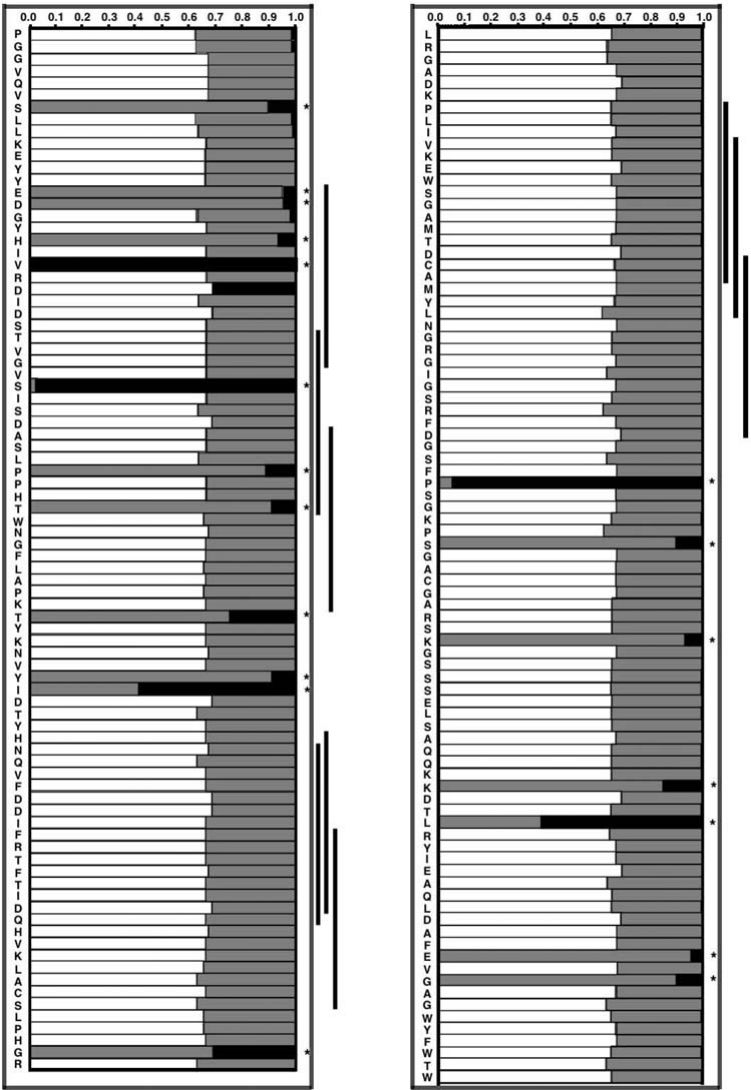
Posterior probabilities showed by each site in the exon 2 of the *PBGP43* gene belonging to site classes with different selective pressures (of 18.20 [black], 1.58 [gray], and 0.00 [white bars]) under the free-ratio model. The gp43 amino acid sequence is shown to the left. Sites with a posterior probability higher than 95% have a greater than 1 and are indicated by an asterisk (*). The underlined parts correspond to the regions that according to SYFPEITHI prediction are potential epitopes.

Models of positive selection (discrete, selection, beta and ω models) that allow for sites with ω greater than 1 fit the *gp43* data significantly better than the corresponding neutral models (one-ratio, neutral and beta models) (Table 5). Posterior probabilities, as revealed by the discrete model, indicate that the *gp43* codons belong to one of the three classes with different selective pressures, as indicated by the beta and ω model (Figure 3). Using the Bayesian Empirical Bayes (BEB) analysis, 19 sites with a posterior probability greater than 95% of having a greater than 1 value were identified. In order to predict potential antigenic determinants for HLA recognition, we used the program SYPFETHI [44]. As illustrated in Figure 3, seven of the sites under positive selection were located as potential epitopes as predicted with SYFPEITHI.

#### *fks*, *p27* and *cdc42*

DNA sequences obtained from 15 individuals showed low levels of polymorphism in *p27* and *cdc42* (*p27*: π_S1_ = 0.00571; π_PS2_ = 0.00206; π_PS3_ = 0.00031; *cdc42*: π_S1_ = 0.00071; π_PS2_ = 0.00006; π_PS3_ = 0.000011). No insertion-deletions were found. In the *fks* case, most of the sequences were retrieved from the NCBI and the polymorphism level was low (*fks*: π_S1_ = 0.000001; π_PS2_ = 0.00006; π_PS3_ = 0.000013).

### Estimation of Time to the Most Recent Common Ancestor (TMRCA)

The TMRCAs for S1 and PS2 were estimated based on genetic variation at the *gp43* locus and seven other nuclear loci. The results showed that the TRMCA for the *gp43* alleles is longer than for any other gene in *P. brasiliensis* (Table 6), indicating that the polymorphism in *gp43* is significantly older than the polymorphism in the other genes (Signed rank test; P<0.01). This constitutes evidence for balancing selection [49],[50]. Additional evidence for the balancing selection hypothesis in *gp43* comes from the haplotype network previously described for this gene, in which several high frequency haplotypes are separated by long branches [7].

**Table 6:** Maximum-Likelihood Estimates (MLE) and the 95% confidence intervals of Time to the Most Recent Common Ancestor (TMRCA).

Gene	Mean	TRMCA	Confidence interval (95%)
		Variance	
***p27***	0.54	0.321	0.324–1.534
***cdc42***	0.489	0.546	0.297–2.672
***gp43***	2.238	7.118	1.5125–3.262
***arf***	0.815	0.836	0.5678–2.5875
***ord***	1.534	2.411	0.89–1.65
***tub***	1.188	1.865	0.8625–1.7625
***ord***	1.618	2.840	0.9375–3.5625
***fks***	0.611	0.471	0.4341–2.7225

Conversely, the TRMCAs for *cdc42*, *p27* and *fks* were significantly lower than the other genes as is expected if a gene is under positive directional selection.

## Discussion

### Identification of putative virulence factors

Comparisons of DNA sequence differences within and between closely related species can give insights into the temporal scales of molecular evolutionary processes, and into selective pressures on different type of loci. In this study, evidence of different types of positive selection acting on the putative virulence factors was obtained from analysis of the ratio between non-synonymous and synonymous substitution rates in coding regions. A comparison of these virulence factors with housekeeping genes in *P. brasiliensis* showed that a higher proportion of virulence genes evolve under positive selection (37.5% vs. 0%), suggesting that at least some of these genes have an adaptive role. Substantial heterogeneity in the mode of evolution was found both among and within the genes investigated in this study. As predicted from previous studies of evolution of virulence factors in other organisms, the 12 putative virulence factors genes identified as having evolved under positive selection have a wide variety of functions (Table 1, Table S1 and Text S1) [27].

This analysis of positive selection using genomic data identified a set of genes that together with data derived from genetic, expression and biochemical essays, provides some insights into the evolution of *P. brasiliensis* virulence. Some of these genes are involved in the escape from immune recognition (*tsa1*, *sod1*). However, this is just one aspect of the ability of a pathogen to successfully invade and colonize its host, and other genes have proven to be important in pathogenesis, such as the case of heat shock genes that are connected to virulence [32]–[34]. Previous studies have suggested that although virulence factors *sensu* Rappleye and Goldman [33] are key factors in pathogenesis, their study as isolated entities does not provide a holistic picture of the evolutionary dynamics of virulence. The results of this study, and others, support the notion that many essential genes participate in complex networks that comprise the molecular basis of virulence, and that their history is shaped by natural selection.

For most of the genes found to be under positive selection (10 out of 12), biochemical and physiological characteristics are known. Only two genes (*p27* and *gp43*) have unknown functions. All the others were classified in four different categories of genes according to their functions: metabolic related genes (*fas2*, *his1*), cell wall related genes (*fks*, *mnn5*, *ags1*), heat shock proteins, detoxification related genes (*tsa1*, *sod1*, *hsp88*) and signal transduction genes (*cdc42*, *cst20*). A detailed biochemical description and information related to these genes is presented in the Text S1.

### M-K and codon analysis of *p27*, *cdc42*, *fks* and *gp43*

*p27*, *cdc42* and *fks* are genes that are depauperate in genetic variation, as is expected for regions in which advantageous amino acid replacements have been fixed by positive selection. Judging by the significant results of the M-K tests, positive selection has played an important role in the history of these three genes and the depletion of genetic variation within *P. brasiliensis* (at these three loci) is a consequence of positive selection.

The M-K test was not significant for *gp43*. This test has proven to be robust because the sites in which synonymous and non-synonymous mutations occur are interspersed, so that they would be similarly affected by genetic drift and changes in geography [20],[45]. In *gp43*, the M-K test was not able to detect positive selected within the *P. brasiliensis* lineage due to the excess of non-synonymous substitutions within and across species. The persistence of non-synonymous intra- and trans-specific *gp43* polymorphisms within and between lineages of the *P. brasiliensis* complex suggests they have been maintained by historical or contemporary selection [51].

Several recent studies have used the power of modern molecular selection analyses to design experiments based on the molecular evolutionary hypothesis [20]. An example of the importance of this kind of study is that immunization with *gp43* epitopes from one isolate would not be expected to be effective against allthe species complex due to the high level of polymorphism in *gp43*. This has profound implications for the development of a *gp43* vaccine and immunotherapy [52].

It is likely that the evolution of putative virulence factors of *P. brasiliensis* has been driven by the interaction between the pathogen and its extracellular environment. However, it remains unclear whether the positive pressure was derived from the environment when the fungus is in its free-living stages, or from the host's immune system. Determining the function and biochemical roles of the proteins encoded by the genes found to be under positive selection in *P. brasiliensis* should shed light on the corresponding selective pressures.

### Conclusions

Molecular evolutionary analysis should facilitate the identification of biologically important genes through the comparison of nucleotide sequences. Although the methods for positive selection used here are not perfect [23], the identification of positively selected proteins offers a good approach for understanding human pathogenic fungi, in which transformation or production of mutants is difficult (McEwen, personal communication). Positive selection in virulence factors might have different outcomes, including: adaptation of a species to optimize the process of infection, to escape host immune response, inhabit different environmental niches, and also lead to functional diversification of members of multi-gene families.

We hope that identifying and cataloging these loci for this and other groups of fungi will provide others with an evolutionary framework for pursuing directed mutation experiments on the specific functional significance of these genes.

## Supporting Information

Table S1 *P. brasiliensis* genes assigned as putative virulence genes by genomic and proteomic studies (10,11). The table includes the biochemical role of the gene product and study that defined each gene as a putative virulence factor in *P. brasiliensis* and constitutes a more expanded version of Table 1.
(0.05 MB XLS)

Table S2 Accession numbers of the nucleotide sequences of the virulence genes that were analyzed in this study.
(0.04 MB XLS)

Text S1 Biochemical information related to these genes under positive selection.
(0.18 MB DOC)
