# Supplementary material for: Evidence for Positive Selection in Putative Virulence Factors within the Paracoccidioides brasiliensis Species Complex
Source: PLoS Negl Trop Dis. 2008 Sep 17;2(9):e296. doi: 10.1371/journal.pntd.0000296 (PMC2553485; doi:10.1371/journal.pntd.0000296)
Supplement: Text S1 — Biochemical information related to these genes under positive selection. (0.18 MB DOC) [file pntd.0000296.s003.doc]

***Metabolism-related genes***. We found two metabolic genes in *P. brasiliensis* that are under positive selection: one involved in lipid metabolism and a second one (HIS1) in aminoacid biosynthesis.

In fungi, fatty acid synthase consists of two multifunctional proteins, the beta and alpha subunits, coded by the genes *fas1* and *fas2* respectively. Five of the seven enzymatic activities reside in the beta subunit (FAS1), while the remaining two activities, beta-ketoacyl synthase and beta-ketoacyl reductase are in the alpha subunit (FAS2) [1]. Disrupting both alleles of the *C. albicans* *fas2* gene suppresses the organism’s ability establish infection in a murine model of systemic candidiasis [2]. Strains in which a single FAS2 allele was disrupted showed the ability to colonize the oral cavity, but the number of cells recovered from infected animals was approximately fivefold less than for the wild type. [3,4]

HIS1, an enzyme involved in the first stage of histidine biosynthesis, encodes an ATP phosphoribosyltransferase, and was also determined to be under positive selection. Purine biosynthesis has been considered a virulence determinant in pathogenic fungi [5,6]. *C. albicans* mutants in nutritional genes involved in amino acid biosynthesis, such as *leu2*, *hem3* [7] or *his1* [8], showed no differences in virulence when compared to wild-type strains. Nevertheless, HIS1 is still considered a virulence factor by Goldman et al. [9] and Felipe et al. [10].

***Cell-Wall related genes.*** Three genes involved in cell wall synthesis are under positive selection pressures: *gls1*, *ags1* and *mnn5*.

AGS1 is responsible for cell wall alpha(1-3)glucan biosynthesis. In *A. fumigatus*, strains with deletions of AGS1 are not defective in virulence [11]. However, in dimorphic fungi, AGS1 was determined to be a bona fide virulence factor [12]. Rappleye [6] dissected the the biochemical mechanism by which α-(1,3)-glucan facilitates the pathogenesis of dimorphic fungi. The reduction in α-(1,3)-glucan levels, through either laboratory passage in *Histoplasma*, *Blastomyces*, and *Paracoccidioides* [13,14,15] or by silencingα-(1,3)-glucan synthase (AGS1) in *H. capsulatum* [12], has no effect on in vitro growth but attenuates virulence in murine respiratory infection models. α-(1,3)-glucan is present in the outermost layer of the *H. capsulatum* yeast cell wall and contributes to pathogenesis by concealing immunostimulatory β-glucans from detection by host phagocytic cells.

FKS1 and FKS2 are alternative subunits of the glucan synthase complex and are responsible for synthesizing 1,3-β-glucan chains, the major structural polymer of the fungal cell wall. In *S. cerevisiae*, it has been demonstrated that the expression of *fks1* predominates during growth under optimal conditions. In contrast, induction of *fks2* expression is induced in response to pheromone, high extracellular CaCl_2_ concentrations, growth on poor carbon sources or in an *fks1* mutant [16]. FKS2 plays a role in the remodeling of the cell wall during the mating process in *S. cerevisiae* [17,18]. It has also been shown for many fungi [14] , including P. brasilieneis [15,19], that virulence of different isolates in experimental murine infection is related to the relative amounts of alpha -glucan to β-glucan present in the cell wall (i.e., the cell wall of avirulent yeast isolates has more β-glucan and less alpha-glucan). The gene encoding β-glucan synthase (*fks*) is down-regulated in response to the macrophage environment in *P. brasiliensis* [19]. The apparent mechanism of β-glucan host protection may be related to its capacity to stimulate the production of several inflammatory mediators, including tumor necrosis factor alpha (TNF-alpha).

*mnn5*, another gene implied in fungal virulence and under positive selection [20,21,22], catalyzes the transfer of mannose to both α-1,2- and α-1,6-mannobiose, and is regulated by the Fe^2+^ concentration. In *C. albicans* , *mnn5*[∆] mutants showed a hypersensitivity to cell wall-damaging agents, reduction of cell wall mannosylphosphate content, impaired hyphal growth on solid media and attenuated virulence in mice [23]. O-linked mannosyl residues attached to proteins were proven necessary for cell wall integrity and normal morphogenesis (at least in *C. albicans*), mediating adhesion and colonization of host tissues [24].

**Heat Shock Proteins and Detoxification Related Genes**. Three genes belonging this category were determined to be under positive selection by our analysis: TSA1, SOD1 and HSP88.

The *tsa1* gene encodes a thiol peroxidase, a type of peroxidases that provide protection against oxidative damage [25]. The gene product, TSA1, is highly induced when *C. neoformans* is grown at 37ºC. tsa1/tsa1 deficient strains showed a significant reduction in their survival when compared to reconstituted and wild-type strains [26,27]. In *S. cerevisiae*, TSA1 is highly expressed when cells with respiratory deficiency are exposed to H_2_O_2_, indicating that TSA1 is an important component of the antioxidant defense in respiratory-deficient [28]. In addition, a link between oxidative and nitrosative stress pathways was demonstrated, since the tsa1/tsa1 mutants were not only sensitive to H_2_O_2_, but also to nitric oxide in vitro [29,30].

SODs (Superoxide Dismutase) constitute the first line of antioxidant defense and are metalloenzymes that catalyze the disproportionation of O^-^ to O^2^ and H_2_0_2_ whereas catalases and glutathione peroxidases subsequently convert H_2_0_2_ to water. Eukaryotes generally have two major forms of SODs that are encoded by distinct genes: the copper–zinc-dependent SOD (*sod1*), localized in the cytosol, and the manganese dependent SOD (*sod2*) found in mitochondria [31,32]. In *C. albicans* cells lacking *sod1* showed attenuated virulence, suggesting a protective role of SOD1 against oxidative stresses imposed by the host [33]. Similar results were obtained in virulence studies carried out with *C. neoformans*. Cox et al. [34] showed that *sod1*/*sod1* mutants had slower intracellular growth when assayed in both murine and human macrophages and its virulence was attenuated in nasal inhalation-infected mice. The deletion of *sod1* gene in *C. neoformans* var. *gattii* had decreased SOD activity and lower virulence, but also had defects in the expression of other defined virulence factors for *C. neoformans*, i.e., laccase, urease and phospholipase [32].

One of the *P. brasiliensis* Heat Shock Proteins, HSP88, was found to be under positive selection as well. In *Neurospora crassa*, transcripts of *hsp88* are present at normal temperature, but they are strongly induced by heat shock [35,36]. Heat shock proteins, including HSP88, are molecular chaperones or ATP-dependent proteases that play important roles in coping with stress-induced denaturation of other proteins [37,38,39,40]. Notably, some authors consider the possibility that although HSPs may be important for yeast-phase growth and dimorphic switching, they are not considered *bona fide* virulence factor candidates because they likely affect the growth of the parasitic phase in vitro as well as in vivo [6]. Nevertheless we did not exclude essential genes from our analysis to not bias our initial sampling scheme.

***Signal Transduction***. We could also identify two genes from the MAPK pathway (*cst20* and *cdc42*) that were under positive selection. Signal transduction pathway genes have been implicated in the dimorphic switch from mycelial growth to yeast [41]. Most of the information about signaling pathways involved in controlling morphological switching comes from studies about pseudohyphal growth in *S. cerevisiae* [42]. Signaling pathways that control the morphological changes in *P. brasiliensis* are still poorly understood, but the involvement of both cyclic AMP (cAMP) and mitogen-activated protein kinase (MAPK) signal transduction pathways have been reported in other dimorphic fungi [41].

In *S. cerevisiae*, the MAPK cascade responsible for cell integrity mediates cell cycle regulation and cell wall synthesis, responding to different signals including temperature, changes in external osmolarity, and mating pheromone. Components of this pathway in *P. brasiliensis* encompass the same functions in the cascade as in other organisms [41]. In *C. albicans,* *cdc42* mutations caused a defect in the budded-to-hypha-form transition in response to various hypha-inducing signals without affecting normal budded growth, strongly supporting the conclusion that CDC42 role is directly related to the morphological transition. CST20 is predicted to interact with a portion of the CDC42 effector domain [43]. Colonies of C. albicans cells for which CST20 was deleted, revealed defects in the lateral formation of mycelia on synthetic solid “Spider” media. However, hyphal development was not impaired in some other media. Cells deleted for CST20 also showed to be less virulent in a mouse model for systemic candidiasis than the wildtype [44].

**REFERENCES**

1. Chirala SS, Kuziora MA, Spector DM, Wakil SJ (1987) Complementation of mutations and nucleotide sequence of FAS1 gene encoding beta subunit of yeast fatty acid synthase. J Biol Chem 262: 4231-4240.

2. Zhao XJ, McElhaney-Feser GE, Sheridan MJ, Broedel SE, Jr., Cihlar RL (1997) Avirulence of Candida albicans FAS2 mutants in a mouse model of systemic candidiasis. Infect Immun 65: 829-832.

3. Naglik JR, Fidel PL, Jr., Odds FC (2008) Animal models of mucosal Candida infection. FEMS Microbiol Lett.

4. Zhao XJ, McElhaney-Feser GE, Bowen WH, Cole MF, Broedel SE, Jr., et al. (1996) Requirement for the Candida albicans FAS2 gene for infection in a rat model of oropharyngeal candidiasis. Microbiology 142 ( Pt 9): 2509-2514.

5. Navarro-Garcia F, Sanchez M, Nombela C, Pla J (2001) Virulence genes in the pathogenic yeast Candida albicans. FEMS Microbiol Rev 25: 245-268.

6. Rappleye CA, Goldman WE (2006) Defining virulence genes in the dimorphic fungi. Annu Rev Microbiol 60: 281-303.

7. Kirsch DR, Whitney RR (1991) Pathogenicity of Candida albicans auxotrophic mutants in experimental infections. Infect Immun 59: 3297-3300.

8. Alonso-Monge R, Navarro-Garcia F, Molero G, Diez-Orejas R, Gustin M, et al. (1999) Role of the mitogen-activated protein kinase Hog1p in morphogenesis and virulence of Candida albicans. J Bacteriol 181: 3058-3068.

9. Goldman GH, dos Reis Marques E, Duarte Ribeiro DC, de Souza Bernardes LA, Quiapin AC, et al. (2003) Expressed sequence tag analysis of the human pathogen Paracoccidioides brasiliensis yeast phase: identification of putative homologues of Candida albicans virulence and pathogenicity genes. Eukaryot Cell 2: 34-48.

10. Felipe MS, Andrade RV, Petrofeza SS, Maranhao AQ, Torres FA, et al. (2003) Transcriptome characterization of the dimorphic and pathogenic fungus Paracoccidioides brasiliensis by EST analysis. Yeast 20: 263-271.

11. Beauvais A, Maubon D, Park S, Morelle W, Tanguy M, et al. (2005) Two alpha(1-3) glucan synthases with different functions in Aspergillus fumigatus. Appl Environ Microbiol 71: 1531-1538.

12. Rappleye CA, Engle JT, Goldman WE (2004) RNA interference in Histoplasma capsulatum demonstrates a role for alpha-(1,3)-glucan in virulence. Mol Microbiol 53: 153-165.

13. Hogan LH, Klein BS (1994) Altered expression of surface alpha-1,3-glucan in genetically related strains of Blastomyces dermatitidis that differ in virulence. Infect Immun 62: 3543-3546.

14. Klimpel KR, Goldman WE (1988) Cell walls from avirulent variants of Histoplasma capsulatum lack alpha-(1,3)-glucan. Infect Immun 56: 2997-3000.

15. San-Blas G, San-Blas F, Serrano LE (1977) Host-parasite relationships in the yeastlike form of Paracoccidioides brasiliensis strain IVIC Pb9. Infect Immun 15: 343-346.

16. Zhao C, Jung US, Garrett-Engele P, Roe T, Cyert MS, et al. (1998) Temperature-induced expression of yeast FKS2 is under the dual control of protein kinase C and calcineurin. Mol Cell Biol 18: 1013-1022.

17. Douglas CM, Foor F, Marrinan JA, Morin N, Nielsen JB, et al. (1994) The Saccharomyces cerevisiae FKS1 (ETG1) gene encodes an integral membrane protein which is a subunit of 1,3-beta-D-glucan synthase. Proc Natl Acad Sci U S A 91: 12907-12911.

18. Mazur P, Morin N, Baginsky W, el-Sherbeini M, Clemas JA, et al. (1995) Differential expression and function of two homologous subunits of yeast 1,3-beta-D-glucan synthase. Mol Cell Biol 15: 5671-5681.

19. Tavares AH, Silva SS, Dantas A, Campos EG, Andrade RV, et al. (2007) Early transcriptional response of Paracoccidioides brasiliensis upon internalization by murine macrophages. Microbes Infect 9: 583-590.

20. Buurman ET, Westwater C, Hube B, Brown AJ, Odds FC, et al. (1998) Molecular analysis of CaMnt1p, a mannosyl transferase important for adhesion and virulence of Candida albicans. Proc Natl Acad Sci U S A 95: 7670-7675.

21. Tavares AH, Silva SS, Bernardes VV, Maranhao AQ, Kyaw CM, et al. (2005) Virulence insights from the Paracoccidioides brasiliensis transcriptome. Genet Mol Res 4: 372-389.

22. Timpel C, Strahl-Bolsinger S, Ziegelbauer K, Ernst JF (1998) Multiple functions of Pmt1p-mediated protein O-mannosylation in the fungal pathogen Candida albicans. J Biol Chem 273: 20837-20846.

23. Bai C, Xu XL, Chan FY, Lee RT, Wang Y (2006) MNN5 encodes an iron-regulated alpha-1,2-mannosyltransferase important for protein glycosylation, cell wall integrity, morphogenesis, and virulence in Candida albicans. Eukaryot Cell 5: 238-247.

24. Timpel C, Zink S, Strahl-Bolsinger S, Schroppel K, Ernst J (2000) Morphogenesis, adhesive properties, and antifungal resistance depend on the Pmt6 protein mannosyltransferase in the fungal pathogen candida albicans. J Bacteriol 182: 3063-3071.

25. Rhee SG, Kang SW, Chang TS, Jeong W, Kim K (2001) Peroxiredoxin, a novel family of peroxidases. IUBMB Life 52: 35-41.

26. Missall TA, Pusateri ME, Lodge JK (2004) Thiol peroxidase is critical for virulence and resistance to nitric oxide and peroxide in the fungal pathogen, Cryptococcus neoformans. Mol Microbiol 51: 1447-1458.

27. Steen BR, Lian T, Zuyderduyn S, MacDonald WK, Marra M, et al. (2002) Temperature-regulated transcription in the pathogenic fungus Cryptococcus neoformans. Genome Res 12: 1386-1400.

28. Demasi AP, Pereira GA, Netto LE (2001) Cytosolic thioredoxin peroxidase I is essential for the antioxidant defense of yeast with dysfunctional mitochondria. FEBS Lett 509: 430-434.

29. Brown SM, Campbell LT, Lodge JK (2007) Cryptococcus neoformans, a fungus under stress. Curr Opin Microbiol 10: 320-325.

30. Missall TA, Lodge JK, McEwen JE (2004) Mechanisms of resistance to oxidative and nitrosative stress: implications for fungal survival in mammalian hosts. Eukaryot Cell 3: 835-846.

31. Fridovich I (1995) Superoxide radical and superoxide dismutases. Annu Rev Biochem 64: 97-112.

32. Narasipura SD, Ault JG, Behr MJ, Chaturvedi V, Chaturvedi S (2003) Characterization of Cu,Zn superoxide dismutase (SOD1) gene knock-out mutant of Cryptococcus neoformans var. gattii: role in biology and virulence. Mol Microbiol 47: 1681-1694.

33. Hwang CS, Rhie GE, Oh JH, Huh WK, Yim HS, et al. (2002) Copper- and zinc-containing superoxide dismutase (Cu/ZnSOD) is required for the protection of Candida albicans against oxidative stresses and the expression of its full virulence. Microbiology 148: 3705-3713.

34. Cox GM, Harrison TS, McDade HC, Taborda CP, Heinrich G, et al. (2003) Superoxide dismutase influences the virulence of Cryptococcus neoformans by affecting growth within macrophages. Infect Immun 71: 173-180.

35. Burnie JP, Carter TL, Hodgetts SJ, Matthews RC (2006) Fungal heat-shock proteins in human disease. FEMS Microbiol Rev 30: 53-88.

36. Costa M, Borges CL, Bailao AM, Meirelles GV, Mendonca YA, et al. (2007) Transcriptome profiling of Paracoccidioides brasiliensis yeast-phase cells recovered from infected mice brings new insights into fungal response upon host interaction. Microbiology 153: 4194-4207.

37. Anisimova M BJ, Dunn K, Yang Z (2007) Phylogenomic analysis of natural selection pressure in Streptococcus genomes. BMC Evol Biol 7: 154.

38. Feder ME, Hofmann GE (1999) Heat-shock proteins, molecular chaperones, and the stress response: evolutionary and ecological physiology. Annu Rev Physiol 61: 243-282.

39. Plesofsky-Vig N, Brambl R (1998) Characterization of an 88-kDa heat shock protein of Neurospora crassa that interacts with Hsp30. J Biol Chem 273: 11335-11341.

40. Wickner S, Maurizi MR, Gottesman S (1999) Posttranslational quality control: folding, refolding, and degrading proteins. Science 286: 1888-1893.

41. Fernandes L, Araujo MA, Amaral A, Reis VC, Martins NF, et al. (2005) Cell signaling pathways in Paracoccidioides brasiliensis--inferred from comparisons with other fungi. Genet Mol Res 4: 216-231.

42. Borges-Walmsley MI, Walmsley AR (2000) cAMP signalling in pathogenic fungi: control of dimorphic switching and pathogenicity. Trends Microbiol 8: 133-141.

43. Mitchell AP (1998) Dimorphism and virulence in Candida albicans. Curr Opin Microbiol 1: 687-692.

44. Leberer E, Harcus D, Broadbent ID, Clark KL, Dignard D, et al. (1996) Signal transduction through homologs of the Ste20p and Ste7p protein kinases can trigger hyphal formation in the pathogenic fungus Candida albicans. Proc Natl Acad Sci U S A 93: 13217-13222.
